# FFP2 induced breathing resistance does not affect metabolism and well-being during brisk walking and stair climbing - a randomized controlled trial

**DOI:** 10.1186/s12995-024-00428-3

**Published:** 2024-07-29

**Authors:** Tobias Engeroff, Niclas Hartel, Daniel Niederer, Albert Nienhaus, David A. Groneberg, Lutz Vogt

**Affiliations:** 1 Institute of Occupational, Social and Environmental Medicine, Goethe University Frankfurt, Frankfurt am Main, Germany; 2 Institute of Sport Science, University of Wuppertal, Wuppertal, Germany; 3 Institute for Health Service Research in Dermatology and Nursing, University Medical Center Hamburg-Eppendorf, Hamburg, Germany; 4 Institute of Sport Sciences, Department of Sports Medicine and Exercise Physiology, Goethe-University Frankfurt, Frankfurt am Main, Germany; 5 Institute of Occupational, Social and Environmental Medicine, Division Health and Performance, Goethe University Frankfurt, Theodor-Stern-Kai 7, Building 9B, 60590 Frankfurt am Main, Germany

**Keywords:** Deadspace, Hypercapnia, Hypoxia, Carbon dioxide, Blood gas analysis, Spiroergometry, Aerosol, Infection

## Abstract

**Objectives:**

N95 or Type II filtering face pieces (FFP2) are often worn during work hours or on public transportation to prevent airborne infection. The aim of this randomized controlled crossover study is to assess the impact of FFP2 induced breathing resistance on pulmonary function, blood gas values and discomfort during walking and stair climbing.

**Methods:**

*N* = 16 healthy adults (24.8 ± 2.2 years; 10 females, ) participated. Interventions included (1) six minutes of walking in a 16-meter-long hallway (612 m) and (2) eight minutes of stair climbing in a two-story staircase (420 stairs), both with and without a FFP2 (> 48 h wash-out). Spiroergometric data (Ventilation, breathing frequency, tidal volume, oxygen uptake and carbon dioxide exhalation (primary outcome), end tidal carbon dioxide- and oxygen pressure) and self-reported response (Perceived exertion, dyspnoea and pain) were assessed during activities. Blood gas analysis (capillary carbon dioxide- (pCO_2_) (primary outcome) and oxygen partial pressure (pO_2_), pH, lactate and base excess) was measured immediately after cessation of activities. Manipulation effects (FFP2 versus no mask) were tested using repeated measures analyses of variance.

**Results:**

Analysis showed no effect of FFP2 on pCO_2_ or other blood-gas parameters but on carbon dioxide exhalation during walking: (mean 1067, SD 209 ml/min) (mean 1908, SD 426 ml/min) (F(15) = 19.5; *p* < 0.001; η_p_^2^ = 0.566) compared to no mask wearing (mean 1237, SD 173 ml/min; mean 1908, SD 426 ml/min). Ventilation was decreased and dyspnoea was increased by FFP2 during activities. FFP2 led to lower oxygen uptake and lower end tidal oxygen but higher end tidal carbon dioxide during stair climbing.

**Conclusions:**

FFP2 decreased ventilation based on slower breathing patterns and led to limitations in pulmonary gas exchange and increased subjective dyspnoea. However, invasive diagnostics revealed no signs of clinically relevant metabolic effects immediately after everyday physical activities.

## Introduction

During to the Covid-19 pandemic, tight-fitting face masks such as N95 or Type II filtering face pieces (FFP2) were worn during work hours or on public transportation. Although recent evidence indicates that other mouth and nose protection such as surgical masks might be comparable effective in filtering particle emission, FFP2 are increasingly preferred due to their closer fit to the face and clearly defined filter properties [1, 2]. Just as there is a growing body of evidence supporting the efficacy of face masks in limiting the risk of airborne infections [3, 4], there is an ongoing debate about potential side effects due to mask-induced adaptations in respiratory function [5–7].

Experimental studies already indicate that masks might alter breathing mechanics [8]. Current meta-analyses on the effects of face masks report that increased breathing resistance indeed leads to a decrease in pulmonary function (breathing frequency, tidal volume and ventilation) during progressive exercise tests at the point of maximal exhaustion [9–11]. In contrast, the effect on pulmonary function during steady state exercise is less well researched and might depend on factors such as intensity, duration or type of the activity [9, 10]. Beyond the breathing resistance approach, another influencing factor could be exhaled air, which is trapped between face and mask. This air could be re-inhaled and thus leads to an increase in dead space [12]. The volume of air behind the mask contains less oxygen (17%) and more carbon dioxide (3.0%) compared to the ambient air [13]. This added fraction of dead space could lead to changes in the composition of the alveolar air especially at lower levels of energy expenditure [14].

Although multiple meta-analyses evaluated the alterations of pulmonary gas exchange and function on a rapidly growing number of experimental studies [9–11, 15], only a small number of trials included invasive measures such as blood gas analysis to investigate metabolic consequences. Furthermore, most studies investigating the effect of mask wearing on blood gases in realistic settings have not used randomized controlled designs. Whereas two studies reported increased blood pCO2 during healthcare work [16, 17], others were not able to confirm these effects [18, 19]. One randomized controlled study assessed the effect of masks on blood gases during office and laboratory work and detected no effect as well [20].

Due to the limited evidence concerning the effect of FFP2 during activities of daily living and the contradictory results, it is unclear if the aforementioned limitations in respiratory function lead to decreased blood oxygenation, elevated carbon dioxide or other clinically relevant metabolic effects during habitual activities in realistic settings. Furthermore, additional studies are necessary to examine if frequently mentioned negative subjective consequences of mask-wearing [10, 21] are linked to such metabolic changes.

To address the sketched gap of knowledge, we conducted an experimental study on the effects of wearing a FFP2 during walking and stair climbing on spiroergometric data, blood gas analysis outcomes and self-reported response.

We hypothesized that FFP2 respirators have a detrimental effect on (1) carbon dioxide exhalation which (2) leads to increased blood pCO_2_ during both ground walking and stair climbing. Furthermore, we hypothesized that FFP2 wearing has (3) a detrimental effect on other spiroergometric and blood gas analysis outcomes and (4) an effect on subjective response when compared to no mask wearing during walking or stair climbing.

## Methods

### Study design and ethical aspects

This study has a randomized controlled cross-over design and is approved by the ethics committee of the Department of Psychology and Sports Sciences of the Goethe University (2022-55, approved 30/08/2022). The trial was registered a priori (German Register for Clinical Trials, DRKS-ID: DRKS00030085, date of first trial registration 25/08/2022) and conducted in accordance with the ethical standards set down by the declaration of Helsinki with its recent modification of 2013 (Fortaleza) [22].

### Participants

Participants were recruited between August and October 2022 in a university in Germany. Eligibility criteria included being between 18 and 50 years of age with no (medical or psychosocial) contraindication against vigorous physical activity. Due to the experimental design, authors had direct contact to the participants and thus could identify individual participants during data collection. During analysis data was anonymized. Exclusion criteria were cardiovascular-, pulmonary-, or advanced degenerative musculoskeletal diseases, pregnancy and not completely healed musculoskeletal injury (that affect subjective quality of life or physical performance during walking and stair climbing).

Sample size calculations were performed based on an earlier study comparing CO2 kinetics during steady state exercise with a FFP2 and a surgical mask against a no mask control [23]. A calculation based on an effect size of Cohen’s d = 0.39 (Partial η^2^ 0.136) a significance level of 5% and an 80% power resulted in a sample size of at least eleven participants adopting a crossover design with four measurements in repeated measures analysis of variance (rmANOVA). Calculating with a drop-out rate of 20%, a minimum of 13 participants needed to be included in this study.

**Fig. 1:**
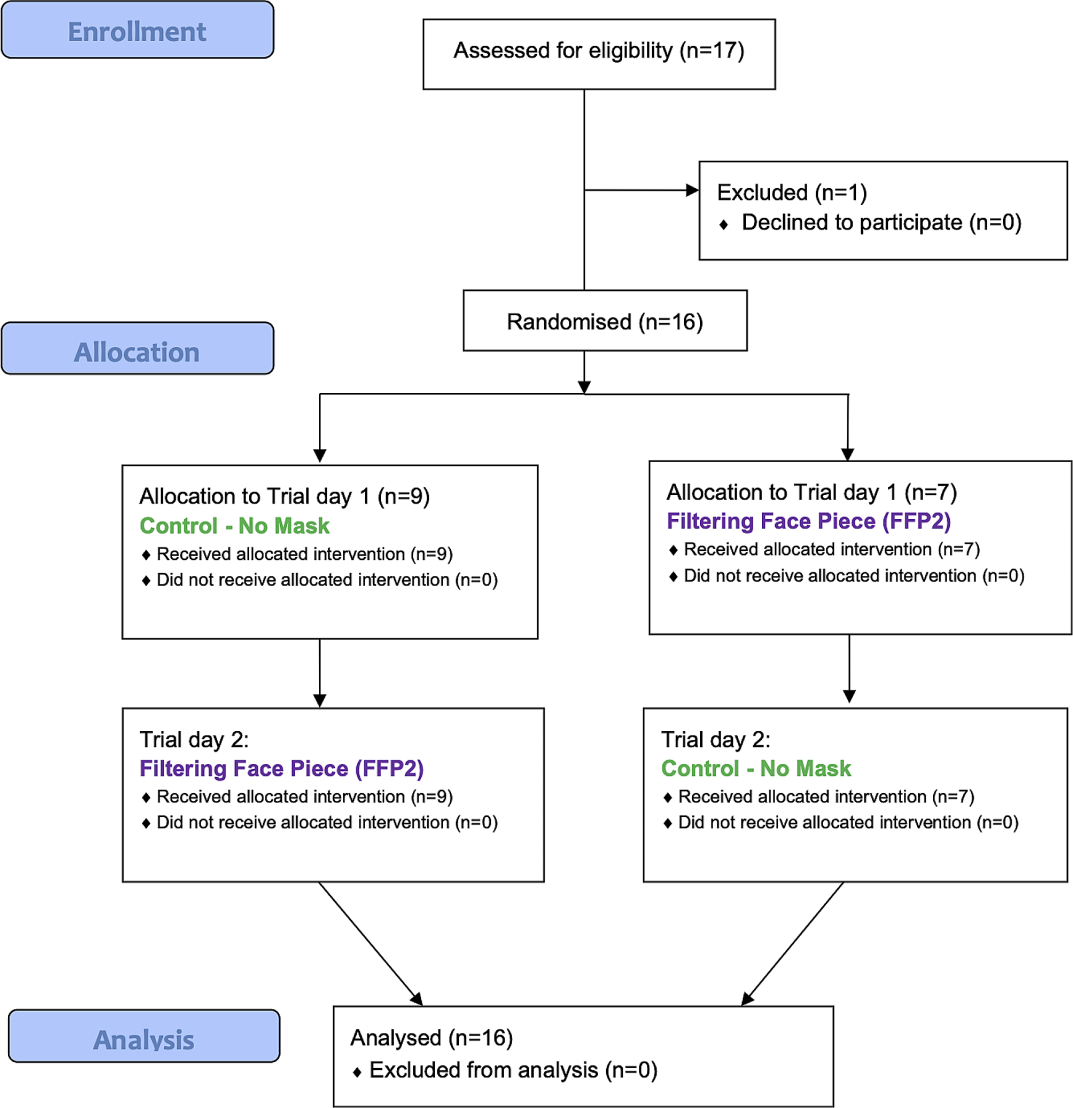
CONSORT flow diagram of the protocol procedures *Figure description: Sixteen participants were assigned to either the FFP2 mask (n = 7) or the unmasked condition (n = 9) by randomisation*,* followed by crossover to the other condition. CONSORT 2010*,* Consolidated Standards of Reporting*

Before study participation, participants were informed on voluntary participation and signed a written informed consent. Eligibility, exclusion and randomization scheme of the protocol is shown in the flow diagram **in** Fig. 1.

### Interventions

All participants performed two interventions types (walking and stair climbing), each once with and once without a fold-flat type FFP2 (IMSTec GmbH, Klein-Winternheim, Germany) on two different trial days separated by a minimum of 48 h. The manipulation (FFP2 or no mask) order was randomized (simple balanced randomization using www.randomizer.org).

Each trial day consisted of a six-minute walking phase, followed by an eight-minute stair climbing phase with ten minutes resting time without wearing a mask in-between. Order allocation was done blinded. The participants were blinded to the respective manipulation until the beginning of each intervention.

Each session was performed at a comparable time of the day (± 2 h) and at days with comparable routines (i.e. working days). Participants had to avoid vigorous physical activities in the 48 h preceding each test and had to maintain their habitual diet behaviour during the timeframe of all three interventions. Furthermore, participants were requested not to take any food or drinks (except for water) in the period of two hours prior to each examination.

At each session, participants first rested for five minutes in a seated position without wearing a FFP2. Baseline data for spiroergometric outcomes, blood gas analysis and subjective response were assessed during this time slot. After that the six minutes ground level walking phase was rolled out in a hallway. The walking speed was standardized and Participants walked 612 m (1,7 m per second). Between the walking and stair climbing intervention, a resting period of ten minutes was held during which the participants were not manipulated (did not wear a mask). Afterwards, the eight minutes stair climbing was performed in on in a two-story staircase. Again, the speed was standardized and all participants completed 24 floors upstairs and downstairs on a standard stairway (stair height 16.5 centimeters, 69.3 m of elevation).

### Outcomes

During baseline testing at each trial day and during the interventions, spiroergometric data and heart rate were measured continuously. Participants self-reported perception was asked every two minutes using standardized scales. The capillary drawings for blood gas analysis were done at the end of baseline testing and immediately after both interventions at the ear lobe with participants in a seated position.

Baseline examination including standard anthropometrical values, educational status (school and study years), habitual physical activity and sedentary behaviour (International Physical Activity Questionnaire IPAQ) [24, 25] took place before the first intervention. Furthermore, participants underwent a step incremental exercise test (7–8 km/h at the start depending on individual fitness; 7° inclination; 0,5 km/h increment every 30 s) on a treadmill until volitional exhaustion to assess maximal oxygen uptake (VO_2_max), maximal heart rate and maximal respiratory exchange ratio. For spiroergometric measures during VO_2_max, baseline and intervention testing a valid and reliable portable analyser was applied (K5, Cosmed, Werneck, Germany) [26].

Baseline data for spiroergometric outcomes, blood gas analysis and subjective response were assessed after five minutes of sitting without a FFP2 at the start of both trial days. Spiroergometric measures during baseline and interventions included ventilation (in litres per minute, l/min), breathing frequency (breaths per minute), tidal volume (in litres, l), oxygen uptake (VO2 in millilitres per minute, ml/min), carbon dioxide exhalation (VCO2 in millilitres per minute, ml/min), respiratory exchange ratio, end tidal carbon dioxide (PetCO2 in millimetres of mercury, mmHg) and end tidal oxygen pressure (PetO2 in millimetres of mercury, mmHg). For blood gas analysis, capillary blood (100 µl) was drawn from an earlobe of the participant and analysed using a validated on-site device (epoc^®^ Blood Analysis System, Epocal Inc., Ottawa, Ontario, Canada) [27]. Outcomes included pH, carbon dioxide partial pressure (pCO2 in millimetres mercury, mmHg), oxygen partial pressure (pO2 in mm/Hg), lactate (mmol per litre, mmol/l) and base excess (BE in mmHg). Subjective response included perceived exertion, based on a 15-point Borg Scale ranging from “very very light” (lowest rating 6) to “very very hard” (highest rating 20) [28], and dyspnoea based on a modified Borg Scale with the same range [29]. Participants were furthermore asked to rate their perception of pain based on a numeric rating scale ranging from “no pain” (lowest rating 0) to “most severe pain imaginable (highest rating 10) [30].

### Data analysis and statistics

We applied Microsoft Excel (Version 16.68) for data processing, Jamovi (Version 2.3.19) for data analysis, and Prism (Version 9) for data presentation. Data were checked for outliers, the distribution was analyzed using box and whisker plots and tested for normal distribution using Shapiro-wilk tests. Descriptive data were reported as means and standard deviations (baseline values and post intervention values) or 95% confidence intervals for all data points available. If datasets were incomplete, missing data was indicated using the number of included datasets in all tables.

Repeated measures analyses of variance (rmANOVA) were applied to analyse the effect of the manipulation (FFP2 versus no mask) on all outcomes and differences between baseline values of both trial days. In a second step, the impact of potential confounders including sex, age, weight, Body-Mass-Index (BMI), physical activity and maximal oxygen uptake capacity was assessed using analysis of covariance (ANCOVA). Pearson correlation was applied to detect associations between subjective measures and spiroergometric or blood gas analysis data which were affected by FFP2 application. We applied Bonferroni corrections for multiple comparisons. Due to 34 between-manipulations-comparisons *p* ≤ 0.001 is considered statistically significant.

## Results

### Demographic and baseline data

Sixteen (16) humans (24.8 ± 2.2 years; 10 females, 6 males) were recruited, participated in this study and completed the study protocol without any adverse event (Fig. 1). Nine participants started without a mask and 7 with the FFP2 condition. Shapiro-Wilk tests showed no significant difference to normal distribution for all datasets. Demographics, including anthropometric, physical activity and physical performance data, are shown in Table 1.

Baseline data of spiroergometric, blood gas and subjective parameters for trials with and without a manipulation (FFP2 and no mask control) are listed in Table 2. No differences between baseline data of both days occurred.

**Table 1:** Demographics, self-reported exercise amounts and exercise capacity of our sample

Outcome / Dimension	Unit	Mean, Standard Deviation
**Anthropometric Data**		
Age	Years	24.8, 2.3
Weight	kg	70.1, 13.4
Height	cm	172.0, 10.2
Body mass index	kg/m^2^	23.4, 2.1
**Physical activity via International Physical Activity Questionnaire**		
Vigorous physical activity	h/week	1.3, 0.5
Moderate physical activity	h/week	1.1, 0.9
Walking	h/week	1.3, 0.8
Sedentary behaviour	h/week	5.1, 2.4
**Incremental exercise test**		
Maximal running speed	kg/h	11.6, 1.4
Maximal heart rate	Beats per minute	191.0, 8.7
Maximal oxygen uptake	ml/min*kg bodyweight	55.0, 9.0
Respiratory exchange ratio		1.2, 0.1

**Table 2:** Descriptive data and analyses of Variance (ANOVAs) results of baseline values for spiroergometric data (ventilatory outcomes, pulmonary gas exchange measures and heart rate), blood gas analysis measures and subjective outcomes

Baseline Data on both trial days			
Outcomes (Units)	Mean, Standard Deviation		ANOVA
	Trial 1 Walk no mask	Trial 2 Walk FFP2	
**Breathing frequency** **(Breaths per minute)**	16.00, 4.00	16.00, 3.00	*p* = 0.891, n^2^ = 0.001, F(15) = 0.019
**Tidal volume** **(Liter)**	0.70, 0.19	0.74, 0.23	*p* = 0.539, n^2^ = 0.026, F(15) = 0.395
**Ventilation** **(Liter per minute)**	10.70, 2.81	11.10, 2.18	*p* = 0.561, n^2^ = 0.023, F(15) = 0.354
**Heart rate** **(beats per minute)**	79.00, 15.00	77.00, 14.00	*p* = 0.459, n^2^ = 0.037, F(15) = 0.577
**Oxygen uptake** **(Milliliters per minute)**	356.00, 91.80	374.57, 90.04	*p* = 0.530, n^2^ = 0.027, F(15) = 0.413
**Carbon dioxide exhalation** **(Milliliters per minute)**	297.00, 72.20	305.00, 60.40	*p* = 0.734, n^2^ = 0.008, F(15) = 0.120
**Respiratory exchange ratio**	0.84, 0.07	0.83, 0.07	*p* = 0.483, n^2^ = 0.033, F(15) = 0.516
**End tidal oxygen pressure** **(Millimeters of mercury)**	110.00, 3.97	110.00, 3.79	*p* = 0.708, n^2^ = 0.010, F(15) = 0.146
**End tidal carbon dioxide pressure** **(Millimeters of mercury)**	33.80, 3.17	32.90, 2.82	*p* = 0.283, n^2^ = 0.076, F(15) = 1.24
**Oxygen partial pressure** **(Millimeters of mercury)**	73.80, 5.85	76.70, 8.89	*p* = 0.166, n^2^ = 0.124, F(15) = 2.12
**Carbon dioxide partial pressure** **(Millimeters of mercury)**	37.60, 3.18	37.10, 4.01	*p* = 0.439, n^2^ = 0.040, F(15) = 0.633
**pH value**	7.42, 0.02	7.43, 0.02	*p* = 0.297, n^2^ = 0.072, F(15) = 1.17
**Base Excess** **(Millimoles per liter)**	0.20, 2.01	0.23, 2.04	*p* = 0.894, n^2^ = 0.001, F(15) = 0.019
**Laktate** **(Millimoles per liter)**	0.97, 0.31	1.00, 0.29	*p* = 0.686, n^2^ = 0.011, F(15) = 0.170
**Perceived exertion** **(Range 6–20)**	6.56, 1.09	6.69, 0.79	*p* = 0.633, n^2^ = 0.016, F(15) = 0.238
**Perceived dyspnoea** **(Range 6–20)**	7.19, 1.33	7.50, 1.51	*p* = 0.484, n^2^ = 0.033, F(15) = 0.516
**Perceived pain** **(Range 1–10)**	1.25, 1.00	1.31, 0.70	*p* = 0.849, n^2^ = 0.003, F(15) = 0.038

### Spiroergometric data

**Fig. 2:**
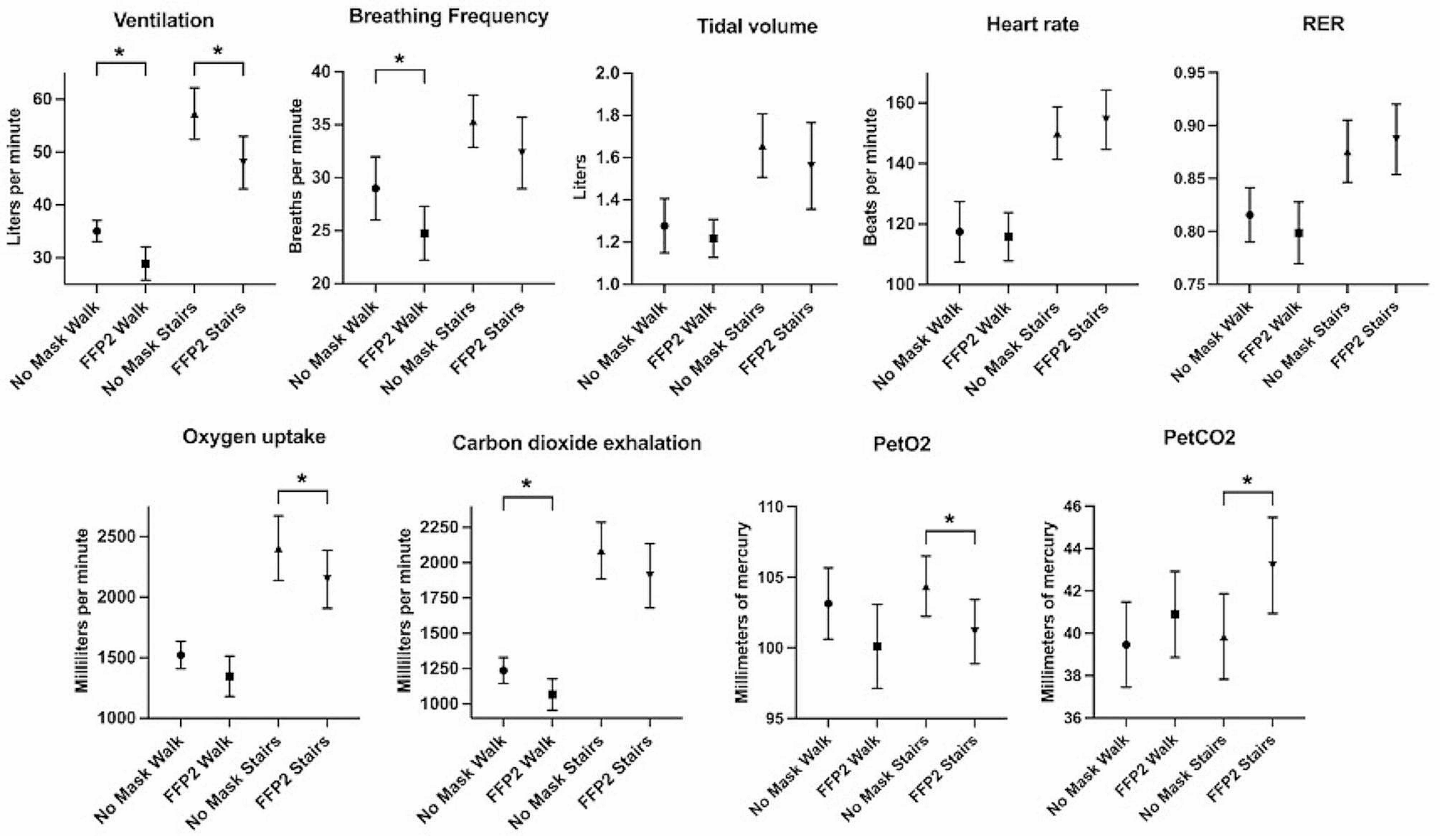
Mean values and 95% confidence intervals of spiroergometric data *Figure description: Indicated are ventilation*,* breathing frequency*,* tidal volume*,* heart rate*,* respiratory exchange ratio (RER) oxygen uptake and carbon dioxide exhalation (primary outcome)*, end tidal carbon dioxide- (petCO2) and oxygen pressure(petO2) during the walking and the stair climbing condition with no mask and a FFP2 mask. Significant ANOVA results are indicated with an asterisk (*)

Descriptive data and the detailed results of the between-manipulations-comparisons for the spiroergometric data of the walking and stair climbing phase are depicted in Table 3. Wearing a FFP2 decreased breathing frequency, ventilation and carbon dioxide exhalation during walking. During stair climbing, ventilation, oxygen uptake and end tidal pressures for oxygen and carbon dioxide were affected by FFP2. Furthermore, FFP2 wearing led to a tendency for lower oxygen uptake and end tidal oxygen pressure during walking and a tendency for decreased breathing frequency during stair climbing. Heart rate and tidal volume during both activities were not affected by mask wearing. Figure 2 shows 95% confidence intervals of spiroergometric outcomes. Analysis of covariance revealed a significant interaction between VO_2_max and the effect of FFP2 wearing on oxygen uptake (F(1)=10.309; p=0.006) and carbon dioxide exhalation (F(1)=5.597; p=0.033) during stair climbing. Other covariates (sex, age, weight, BMI, physical activity) showed no significant interaction with between-manipulations effects.

### Blood gas analysis and self-reported outcomes

**Fig. 3:**
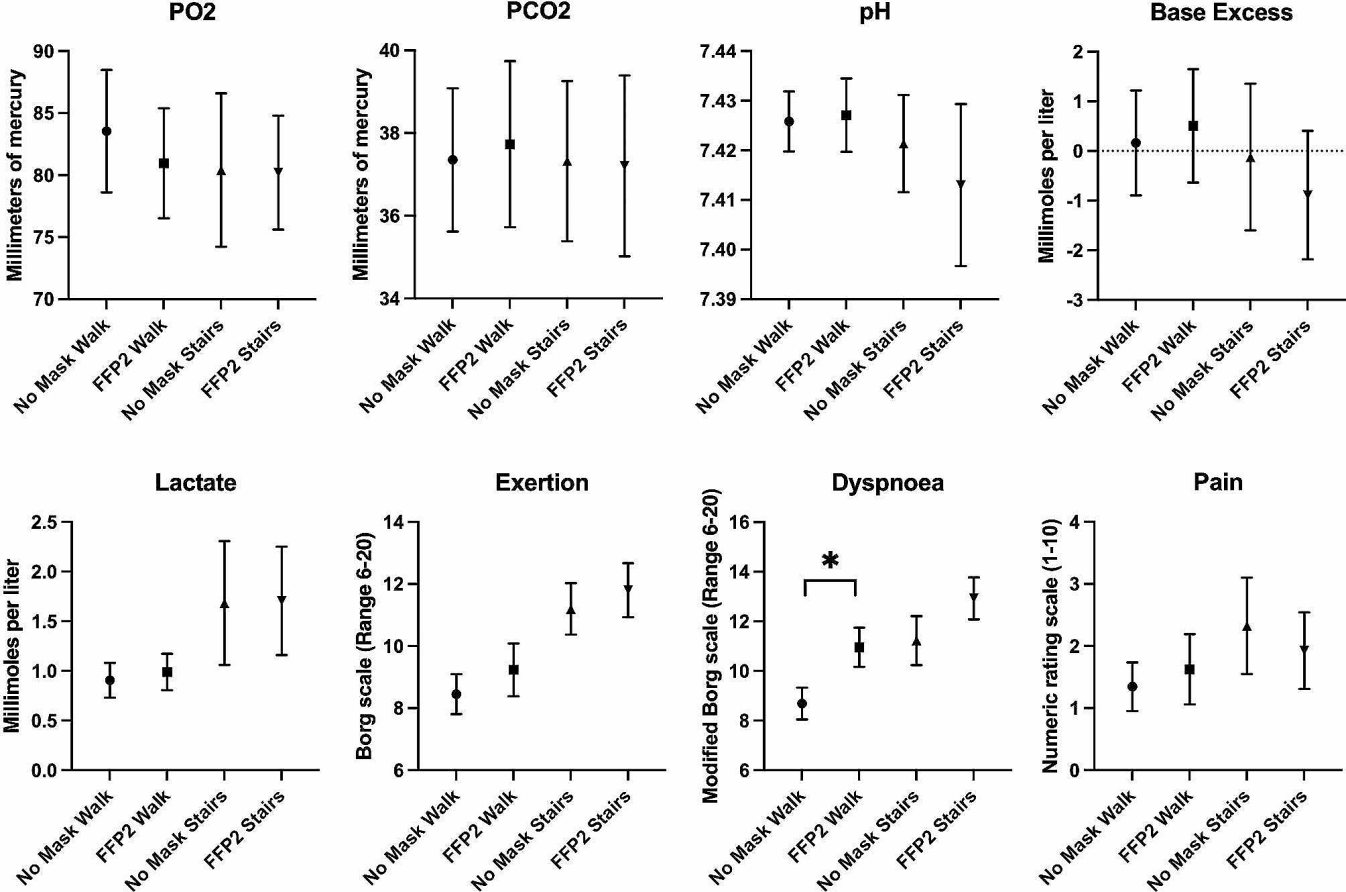
Mean values and 95% confidence intervals of blood gas analysis outcomes *Figure description: Indicated are capillary carbon dioxide- (PCO2) (primary outcome) and oxygen partial pressure (PO2)*,* pH*,* lactate*,* base excess and perceived exertion*,* perceived dyspnoea*, pain during the walking and the stair climbing condition with no mask and a FFP2 mask. Significant ANOVA results are indicated with an asterisk (*)

Descriptive data and the detailed results of the between-manipulations-comparisons for blood gas analysis outcomes and all subjective outcomes of the walking and stair climbing phase are depicted in Table 3. Figure 3 shows 95% confidence intervals of blood gas analysis outcomes. Although there were notable differences between baseline and post intervention values for base excess and lactate, blood gas analysis outcomes after physical activities with and without a mask were in a comparable range and ANOVAs showed no significant between-manipulations-differences.

Participants perceived more dyspnoea during walking when a FFP2 was applied. Figure 3 shows 95% confidence intervals of self-report outcomes. No significant interaction with any tested covariates occurred. Mean values of perceived exertion were higher during stair climbing than during walking but ANOVAs indicated no effect of FFP2 wearing on exertion or pain during both physical activities. Pearson correlation of perceived dyspnoea with spiroergometric outcomes and covariates showed no significant associations.

**Table 3:** Descriptive data and analyses of Variance (ANOVAs) results for between-manipulations-comparisons (FFP2 vs. no mask) for spiroergometric data (ventilatory outcomes, pulmonary gas exchange measures and heart rate), blood gas analysis measures and subjective outcomes during walking and stair climbing. *indicates significant between-manipulations-effects

Between-Manipulations-Comparisons						
Outcomes (Units)	Walking			Stair Climbing		
	Mean, Standard Deviation		ANOVA	Mean, Standard Deviation		ANOVA
	**No mask**	**FFP2**		**No mask**	**FFP2**	
**Breathing frequency** **(Breaths per minute)**	29.00, 5.60	24.80, 4.77	*p* < 0.001* n^2^ = 0.658 F(15) = 28.9	35.90, 4.63	32.30, 6.34	*p* = 0.04 n^2^ = 0.429 F(15) = 11.2
**Tidal volume (Liter)**	1.28, 0.24	1.22, 0.17	*p* = 0.22 n^2^ = 0.1 F(15) = 1.65	1.66, 0.28	1.56, 0.38	*p* = 0.078 n^2^ = 0.192 F(15) = 3.57
**Ventilation** **(Liter per minute)**	35.10, 3.77	28.90, 5.88	*p* < 0.001* n^2^ = 0.714 F(15) = 37.4	57.30, 9.13	48.00, 9.35	*p* < 0.001* n^2^ = 0.88 F(15) = 110
**Heart rate** **(Beats per minute)**	117.00, 18.80	116.00, 15.00	*p* = 0.488 n^2^ = 0.033 F(15) = 0.51	150.00, 16.30	155.00, 18.30	*p* = 0.065 n^2^ = 0.209 F(15) = 3.96
**Oxygen uptake** **(Milliliters per minute)**	1522, 211	1347, 313	*p* = 0.013 n^2^ = 0.348 F(15) = 8.02	2405, 501	2148, 450	*p* < 0.001* n^2^ = 0.685 F(15) = 32.6
**Carbon dioxide exhalation** **(Milliliters per minute)**	1237, 173	1067, 209	*p* < 0.001* n^2^ = 0.566 F(15) = 19.5	2087, 376	1908, 426	*p* = 0.002 n^2^ = 0.489 F(15) = 14.3
**Respiratory exchange ratio**	0.82, 0.05	0.80, 0.05	*p* = 0.381 n^2^ = 0.051 F(15) = 0.814	0.88, 0.06	0.89, 0.06	*p* = 0.538 n^2^ = 0.026 F(15) = 0.397
**End tidal oxygen pressure** **(Millimeters of mercury)**	103.00, 4.73	100.00, 5.58	*p* = 0.024 n^2^ = 0.297 F(15) = 6.33	104.00, 4.01	101.00, 4.27	*p* < 0.001* n^2^ = 0.817 F(15) = 67.2
**End tidal carbon dioxide pressure** **(Millimeters of mercury)**	39.50, 3.78	40.90, 3.81	*p* = 0.0511 n^2^ = 0.230 F(15) = 4.49	39.80, 3.79	43.20, 4.25	*p* < 0.001* n^2^ = 0.569 F(15) = 19.8
**Oxygen partial pressure** **(Millimeters of mercury)**	83.50, 9.26 (*n* = 16)	81.00, 7.68 (*n* = 14)	*p* = 0.179 n^2^ = 0.125 F(14) = 2.00	80.40, 10.70 (*n* = 14)	80.20, 7.95 (*n* = 14)	*p* = 0.994 n^2^ = 0.000 F(13) = 0.00006
**Carbon dioxide partial pressure** **(Millimeters of mercury)**	37.40, 3.25 (*n* = 16)	37.70, 3.48 (*n* = 14)	*p* = 0.575 n^2^ = 0.025 F(13) = 0.330	37.30, 3.35 (*n* = 14)	37.20, 4.10 (*n* = 16)	*p* = 0.4111 n^2^ = 0.053 F(13) = 0.721
**pH value**	7.43, 0.01 (*n* = 16)	7.43, 0.01 (*n* = 14)	*p* = 0.973 n^2^ = 0.000 F(13) = 0.001	7.42, 0.02 (*n* = 14)	7.41, 0.03 (*n* = 16)	*P* = 0.148 n^2^ = 0.156 F(13) = 2.27
**Base Excess** **(Millimoles per liter)**	0.16, 1.98 (*n* = 16)	0.51, 1.98 (*n* = 14)	*p* = 0.609 n^2^ = 0.021 F(13) = 0.274	-0.12, 2.56 (*n* = 14)	-0.89, 2.42 (*n* = 16)	*p* = 0.114 n^2^ = 0.181 F(13) = 2.88
**Lactate** **(Millimoles per liter)**	0.91, 0.33 (*n* = 16)	0.99, 0.32 (*n* = 14)	*p* = 0.346 n^2^ = 0.069 F(13) = 0.958	1.68, 1.08 (*n* = 14)	1.71, 1.02 (*n* = 16)	*p* = 0.787 n^2^ = 0.006 F(13) = 0.076
**Perceived exertion** **(Range 6–20)**	8.45, 1.21	9.23, 1.60	*p* = 0.074 n^2^ = 0.197 F(15) = 3.68	11.20, 1.56	11.80, 1.63	*p* = 0.142 n^2^ = 0.138 F(15) = 2.40
**Perceived dyspnoea** **(Range 6–20)**	8.69, 1.20	11.00, 1.48	*p* < 0.001* n^2^ = 0.605 F(15) = 22.9	11.20, 1.85	12.90, 1.58	*P* = 0.003 n^2^ = 0.462 F(15) = 12.9
**Perceived pain** **(Range 1–10)**	1.34, 0.74	1.63, 1.06	*p* = 0.191 n^2^ = 0.111 F(15) = 1.88	2.33, 1.45	1.93, 1.16	*p* = 0.120 n^2^ = 0.153 F(15) = 2.71

## Discussion

### Hypotheses verification

We found a decrease in ventilation and breathing frequency but no significant decrease in tidal volume during walking and stair climbing when an FFP2 mask is worn. These alterations seem to effect carbon dioxide exhalation during both activities and oxygen uptake during stair climbing. Our first hypothesis thus can be verified. However, our data showed no impact of FFP2 wearing on blood carbon dioxide or oxygen levels immediately after both exercise interventions ended. This leads to a rejection of our second hypothesis. Against our third hypothesis, other invasive markers of clinically relevant metabolic effects remained unaffected as well. Participants perceived comparable activity-related exertion but more dyspnoea when a FFP2 was worn during walking. Hypothesis four thus can partly be verified. Increased dyspnoea was not directly associated with the mask induced decrease in ventilatory performance or pulmonary gas exchange.

### Mechanisms and effect discussion

So far only a limited number of studies applied invasive blood gas analysis to assess the metabolic impact of FFP2 wearing. Five of these studies analyzed the impact during physical inactivity and reported no alterations of oxygen partial pressure (pO2), carbon dioxide (pCO2), pH, base excess and lactate concentration in healthy subjects [23, 31–33] and patients with stable chronic heart failure [34]. During this metabolic state adaptations in breathing patterns such as increased tidal volume are thus likely to compensate the influences of increased breathing resistance and additional dead space [9, 23, 31, 32]. Five studies report an effect on capillary blood pCO2 during steady state ergometer cycling with moderate and vigorous intensity [23, 35] as well as during maximal workload [20, 36, 37]. Contrastingly two studies on healthy subjects [33] and patients chronic heart failure [34] showed no effects during maximal workload. Two randomized controlled studies assessed the effect of surgical masks and FFP2 during low intensity exercise [38, 39]. Whereas Michalik and colleagues reported an effect of mask wearing on lactate concentrations [38], Vinettis group detected increased pCO2 levels during ergometer cycling with a mask but no changes in lactate concentration [39].

In consideration of this inconclusive evidence, the most relevant novelty of our study is the combination of spiroergometric measurements and invasive blood gas analysis during low to moderate intensity physical activity. So far only three studies combined breathing gas- and blood gas analyses. Two compared the effects of surgical masks and FFP2 to a no-mask control condition at the point of maximal exhaustion during an incremental exercise test [33, 34]. In both studies, Fikenzer and colleagues reported significant effects on ventilation and oxygen uptake whereas blood gas analyses revealed no resulting alterations in capillary pO_2_, pCO_2_ or pH in healthy participants [33] and patients with heart failure [34]. A third study confirmed an impact on breathing performance, pulmonal gas exchange and blood carbon dioxide partial pressure during ergometer cycling with moderate and vigorous intensity [35]. We extend these findings not only by assessing low to moderate intensity but also by analysing carbon dioxide exhalation and end tidal O_2_ and CO_2_ pressure. Using this approach, we are able to confirm that decreased ventilation leads to differences in end tidal pressure and gas exchange even during low to moderate intensity physical activity. Furthermore, our data indicates that physical fitness has an impact on FFP2 induced alterations of oxygen uptake and carbon dioxide exhalation during moderate intensity physical activity. In line with earlier research on exhaustive exercise [33, 34] and physical rest [23, 31–33, 39], we confirm that the limitations in pulmonary gas exchange during exercise seem to be rather small and do not affect capillary pCO_2_ and pO_2_ immediately after exercise cessation. In contrast to these findings, one study on vigorous intensity steady state exercise in healthy subjects [23] and one study on progressive exercise until exhaustion in patients with coronary artery diseases or hypertension [36] included blood gas analysis during exercise but no breathing gas measurements and were able to detect significant effects on pCO_2_ and corresponding differences in pH, base excess and hydrogen carbonate [23, 36]. Overall, current evidence indicates that these metabolic effects might be linked to exercise intensity and are likely to be compensated quickly after the end of the activity. Although the differences in methodology between studies with [33, 34] and without [23, 36] spiroergometric testing seem obvious, it is unclear if a tighter fit of FFP2 masks due to the combination with rubber masks or discrepancies of the blood sampling time points could have influenced the results.

The tight fit of the rubber mask over the FFP2 might lead to increased breathing resistance and could reduce the space between FFP2 and face. Another potential problem is an air-leakage between facial skin, protective mask and rubber spiroergometry mask. In a direct response letter to the first experiment of Fikenzer and colleagues [33], a group of experts discussed that, based on the assumption of linear relationships between power output, oxygen consumption and cardiac output, gross ventilation might has been underestimated due to air leakage during expiration [40]. Within our study, the power output between the masked and unmasked exercise condition was matched and the mean heart rate was in a comparable range. Anyhow, our data also show significantly lower ventilation, oxygen uptake and carbon dioxide exhalation. These changes might be overestimated due to the described limitations. In order to control potential problems with ventilation-based measures (tidal volume and ventilation), we additionally analysed end tidal concentrations of oxygen and carbon dioxide and were able to detect significant effects of FFP2 application on both outcomes. Even assuming an undetected measurement error due to leakage, the changes in oxygen and carbon dioxide content in the exhaled air imply a decrease in ventilation in the masked exercise condition. Our data indicates that these changes in pulmonary gas exchange during low to moderate intensity physical activity have no effect on blood gases or other metabolic outcomes if the sampling was done immediately after exercise cessation. Looking at the origin and magnitude of the observed changes, it thus can be concluded that it is rather a limitation of CO_2_ elimination than an increased production due to alterations in energy metabolism. Invasive blood gas analysis was not succesfull during one trial arm for two particpants. Analysis of variance was thus rolled out with all datasets available. Despite the a priori sample size calculation based on a study on the impact on CO2 kinetics [23], our study may underestimate the effect on other metabolic parameters due to the rather small sample size. Our analysis indicates the influence of FFP2 masks on healthy humans. Further studies are necessary to rule out possible negative influences in individuals with chronic illnesses such as chronic pulmonary diseases, high blood pressure, heart failure or vascular diseases.

In line with current evidence [10], participants in our experiment perceived increased dyspnoea during walking and stair climbing when a FFP2 was worn. Our results confirm meta-analytic data which evaluated the effects of different mask types and indicated that although surgical masks have an effect on perceived exertion, a similar effect does not occur with FFP2 [10]. It is discussed, that the materials and construction of surgical masks might induce more discomfort due to mask suction and deformation during intense breathing [36]. Against earlier findings on a linear relation of perceived exertion with breathing frequency and heart rate [41], we found no association between the subjective and objective effects of FFP2 wearing.

### Practical implications for everyday life and research

FFP2 can be applied by healthy humans during inactivity and habitual physical activities such as climbing multiple flights of stairs or walking a few hundred meters indoors without risking harmful metabolic changes. Therefore, mask wearing can be recommended for people without chronic diseases in order to limit the risk of airborne infection for the general population in settings like public transport, medical facilities or public places. Public information campaigns should highlight that perceived discomfort or light dyspnoea is not necessarily linked to negative or even harmful metabolic effects of protective masks.

Future studies need to evaluate if higher carbon dioxide levels, higher cardiac output or increased breathing resistance during more intense or prolonged physical activities such as structured exercise or physical labour could lead to a detrimental metabolic response or counteract beneficial exercise effects.

## Conclusion

Face masks such as FFP2 induce small changes in pulmonary function and gas exchange during low to moderate intensity physical activity. Invasive metabolic parameters, blood carbon dioxide and oxygen values were in a physiological range and did not affect subjective wellbeing. Healthy adults thus seem to physiologically compensate the impact of FFP2 during physical activities with low to moderate intensity. Consequently, our data underlines that mask wearing in most settings without the option to maintain social distancing over a limited timeframe does not lead to detrimental health consequences in healthy adults. Further studies need to assess the effects of prolonged and repeated mask application especially in real-life settings (shared workspace, long distance public transport) and during activities with up to vigorous intensity (physical labour in crowded areas).

## Data Availability

Data are available upon reasonable request per institutional policy (Contact: Tobias Engeroff, engeroff@sport.uni-frankfurt.de).
